# Heart rate trajectories in patients recovering from acute myocardial infarction: A longitudinal analysis of Apple Watch heart rate recordings

**DOI:** 10.1016/j.cvdhj.2021.05.003

**Published:** 2021-05-12

**Authors:** Daniel Weng, Jie Ding, Apurva Sharma, Lisa Yanek, Helen Xun, Erin M. Spaulding, Ngozi Osuji, Pauline P. Huynh, Oluseye Ogunmoroti, Matthias A. Lee, Ryan Demo, Francoise A. Marvel, Seth S. Martin

**Affiliations:** ∗ Johns Hopkins University School of Medicine, Baltimore, Maryland; † Ciccarone Center for the Prevention of Cardiovascular Disease, Division of Cardiology, Department of Medicine, Johns Hopkins University School of Medicine, Baltimore, Maryland; ‡ Division of Cardiology, Department of Medicine, Johns Hopkins University School of Medicine, Baltimore, Maryland; § Department of Medicine, Johns Hopkins University School of Medicine, Biostatistics, Epidemiology, and Data Management Core Faculty, Baltimore, Maryland; ‖ Johns Hopkins University School of Nursing, Baltimore, Maryland; ¶ Johns Hopkins University Bloomberg School of Public Health, Baltimore, Maryland; # Department of Medicine, Johns Hopkins University School of Medicine, Baltimore, Maryland; ∗∗ Johns Hopkins University Whiting School of Engineering, Baltimore, Maryland

**Keywords:** Apple Watch, Biometrics, Cardiac rehab, Digital health, Heart attack, Heart rate, Mobile health, Myocardial infarction, Remote monitoring, Wearable

## Abstract

**Background:**

Using mobile health, vital signs such as heart rate (HR) can be used to assess a patient’s recovery process from acute events including acute myocardial infarction (AMI).

**Objective:**

We aimed to characterize clinical correlates associated with HR change in the subacute period among patients recovering from AMI.

**Methods:**

HR measurements were collected from 91 patients (4447 HR recordings) enrolled in the MiCORE study using the Apple Watch and Corrie smartphone application. Mixed regression models were used to estimate the associations of patient-level characteristics during hospital admission with HR changes over 30 days postdischarge.

**Results:**

The mean daily HR at admission was 78.0 beats per minute (bpm) (95% confidence interval 76.1 to 79.8), declining 0.2 bpm/day (-0.3 to -0.1) under a linear model of HR change. History of coronary artery bypass graft, history of depression, or being discharged on anticoagulants was associated with a higher admission HR. Having a history of hypertension, type 2 diabetes mellitus (T2DM), or hyperlipidemia was associated with a slower decrease in HR over time, but not with HR during admission.

**Conclusion:**

While a declining HR was observed in AMI patients over 30 days postdischarge, patients with hypertension, T2DM, or hyperlipidemia showed a slower decrease in HR relative to their counterparts. This study demonstrates the feasibility of using wearables to model the recovery process of patients with AMI and represents a first step in helping pinpoint patients vulnerable to decompensation.

## Graphical abstract

**Figure:**
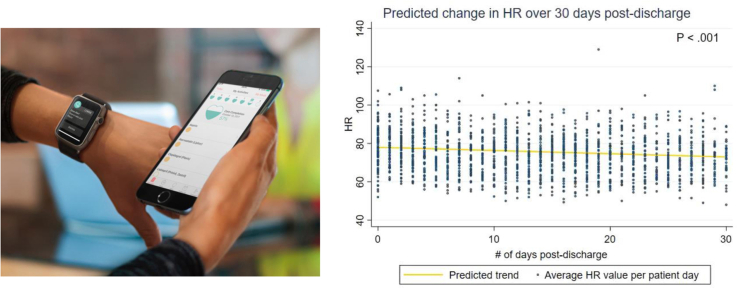

**Key Findings**

• Patients recovering from AMI showed a small progressive decline in mean daily HR (-0.2 bpm/day) over 30 days post-discharge.
• Among recovering patients, patients with diagnosed depression, prior CABG before index admission, and those discharged with diuretics or anticoagulants demonstrated a higher mean admission HR compared to patients without such characteristics.
• Patients with a history of T2DM, hypertension, or hyperlipidemia had a slower decrease in HR compared to patients without those conditions.
• 11 patients were readmitted within 30 days, with a mean time to readmission of 6.9 days after discharge. Of these patients, 6 had HR recordings >90 bpm within 3 days prior to readmission.

## Introduction

Acute myocardial infarction (AMI) is a leading cause of morbidity and mortality in the United States. AMI accounts for more than 600,000 annual hospitalizations, and 1 in 6 patients are readmitted within 30 days postdischarge.^1^ Given the burden of AMI readmissions on the healthcare system, research has focused on determining relevant predictors of poor AMI recovery to guide interventions, 1 of which is elevated resting heart rate (HR).^2^, ^3^, ^4^, ^5^ Elevated resting HRs, defined as a HR >70 beats per minute (bpm), has been associated with higher morbidity and mortality for patients with acute coronary syndrome both at 1-month and 1-year follow-up.^2^, ^3^, ^4^, ^5^ The utility of these studies may be limited, however, owing to the snapshot nature of the HR data obtained in ambulatory settings, and continuous monitoring being restricted to the hospital setting. There is a paucity of research examining HRs for recovering AMI patients in their home settings.

With the advent of personal wearables, individuals can now more conveniently monitor their HR multiple times daily. The Apple Watch has been shown to yield a high degree of concordance with electrocardiograms, especially in regard to resting HR, compared to other devices.^6^ This study aimed to (1) use dynamic HR measures collected from the Apple Watch and characterize the 30-day HR trajectories among subacute recovering AMI patients; and (2) identify potential predictors associated with admission HR status and the rate of HR change postdischarge.

## Methods

### Study participants

We used data from a subgroup of patients in the Myocardial infarction COmbined-device Recovery Enhancement (MiCORE) study. MiCORE was a multicenter prospective study assessing the effectiveness of the Corrie Health Digital Platform, consisting of a smartphone app, Apple Watch, and wireless blood pressure cuff in reducing 30-day readmission after AMI.^7^ In brief, Corrie Health offered a suite of features to patients, including interactive educational content, medication reminders, biometric dashboards, appointment reminders, and connection to their healthcare network. The MiCORE study was approved by the Johns Hopkins Medicine Institutional Review Board (IRB0009938). The research reported in this paper adhered to guidelines outlined by the Helsinki Declaration for human research. Patient information was stored in the REDCap (Research Electronic Data Capture) application. Consent was provided by all patients as part of the MiCORE study. Details regarding enrollment can be found in a separate methods publication.^7^

**Figure 1:**
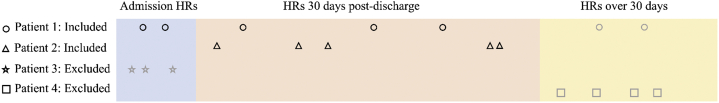
Visual representation of patient and heart rate (HR) inclusion criteria. The y-axis describes 4 sample patients, their associated HR shape, and whether they would be considered included or excluded based on their HR distribution. The x-axis describes the time associated with each HR recording. Each shape represents an individual HR associated with the respective patient. Bolded shapes signify inclusion of the HR, while grayed-out ones were excluded.

Among 200 MiCORE patients who used Corrie, 34 patients were excluded because they participated before backend data collection capabilities were developed. Of the 126 patients with available HR records, patients were included if they had any HRs between first day postdischarge and 30 days postdischarge. HRs were included if they fell in between the patient’s study enrollment during admission and 30 days postdischarge (Figure 1). Thirty-five patients had no HRs within 30 days postdischarge, leaving 91 patients who provided 4447 valid HR measurements (ie, values falling within the range [30–210 bpm] of optical heart sensors^8^). Ten patients included in this study had no admission HRs recorded, 23 patients had 1 admission HR, and 68 had at least 2.

### HR measurements from Apple Watch

**Figure 2:**
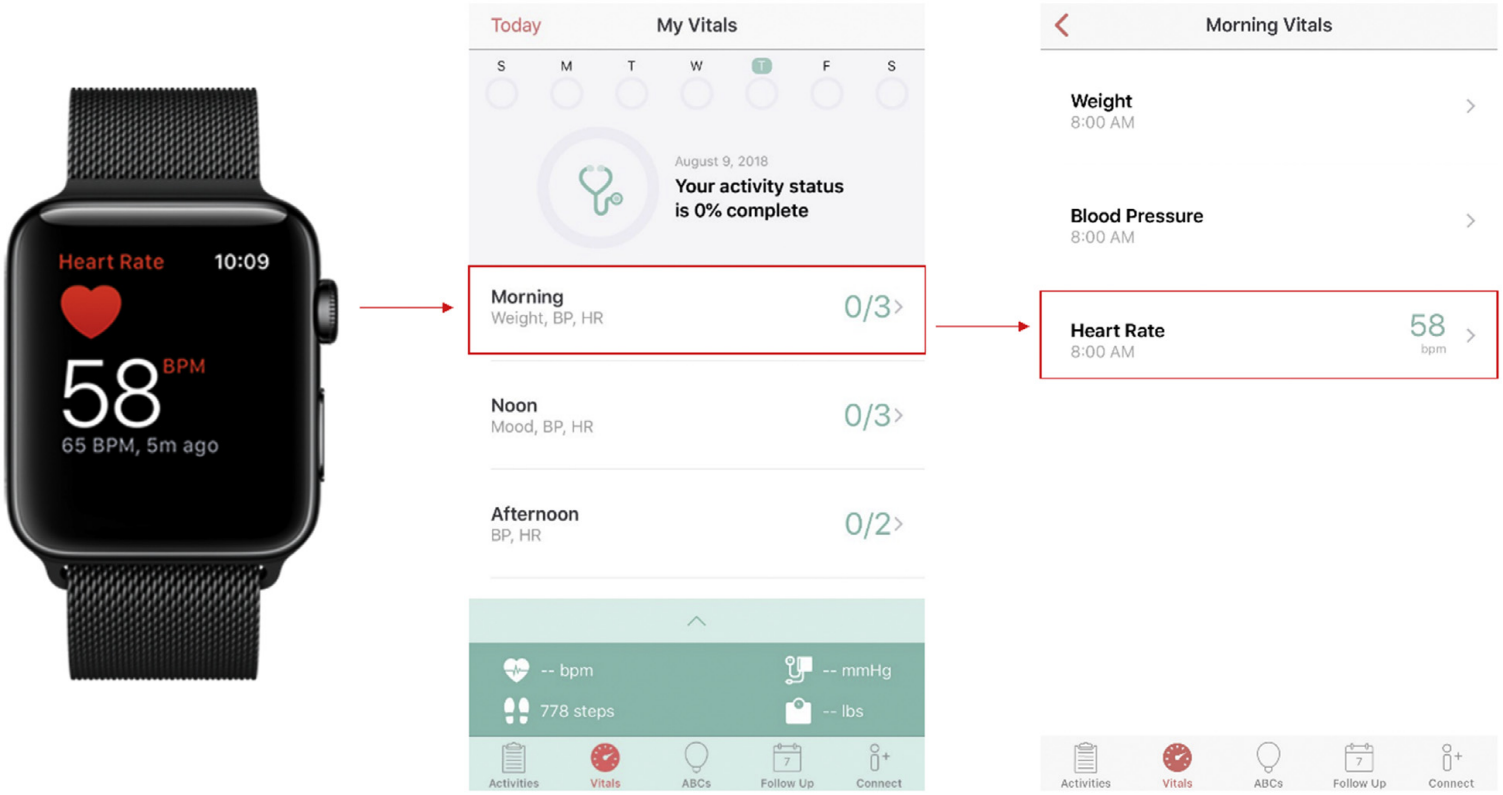
Heart rate recordings—from Apple Watch to the Corrie Health application.

HR measurements were obtained through the Apple Watch Series 1 (Apple Inc, Cupertino, CA) and stored in the Corrie Health Amazon Web Services server, which is compliant with the Health Insurance Portability and Accountability Act. The Corrie Health smartphone app screens individual HRs recorded by the Apple Watch and extracts the first HR recording that satisfies the time bounds. These time bounds were as follows: “Morning” – between 8 AM and 11:59 AM; “Noon” – between 12 PM and 3:59 PM; “Afternoon” – between 4 PM and 7:59 PM; and “Evening” – between 8 PM and 11:59 PM. As demonstrated in Figure 2, a single patient could have a maximum of 4 HR recordings within a given day. An overall estimate of daily HR was further calculated by averaging the morning, noon, afternoon, and evening HRs each day per patient. The time windows were created to increase app engagement (having patients revisit the app a few times a day) and to input manual measurements like mood and weight. HRs that were recorded during the patient’s assumed sleeping period (12 AM – 7:59 AM) were not collected within the designated time windows.

### Clinical characteristics

Clinical characteristics of patients were obtained through the EPIC medical record, including (1) sociodemographic, anthropometric, and lifestyle factors: age, sex, race (white vs nonwhite), smoking status, Medicaid status, and body mass index; (2) admission characteristics, in particular diagnosis; left ventricular ejection fraction (LVEF) less than 40%; revascularization status; whether percutaneous coronary intervention (PCI), coronary artery bypass graft (CABG), or neither was performed; transfusion status; thrombolysis in myocardial infarction (TIMI) major bleeding; development of cardiogenic shock; development of heart failure; (3) history of hyperlipidemia, hypertension, heart failure, previous PCI, previous CABG, stroke, cerebrovascular disease, atrial fibrillation (AFIB), peripheral vascular disease, previous myocardial infarction (MI), type 2 diabetes mellitus (T2DM), chronic lung disease, dialysis, genitourinary and gastrointestinal bleeds in the last 6 months, depression; and (4) discharge medications for MI, specifically adenosine diphosphate receptor inhibitors, angiotensin-converting enzyme inhibitors, angiotensin II receptor blockers, anticoagulants, diuretics, beta blockers (BBs), aspirin, high-intensity statins, and calcium channel blockers (CCBs). All relevant clinical characteristics captured during index admission were defined by ICD-10 codes or through manual chart review if not associated with an ICD-10 code (Supplemental Table 1). Manually reviewed variables include LVEF <40%, incomplete revascularization, whether a PCI or CABG was performed, presence of TIMI major bleeding, and changes to discharge medications. Medicaid status was used as a binary covariate in the model to help control for socioeconomic status and healthcare access. Patient income data were also collected as an optional fill-in; however, most patients did not complete this field. Details regarding primary insurance and racial demographic variables can be found in Supplemental Table 2.

LVEF was obtained from the patient’s first echocardiographic report after study enrollment. Incomplete revascularization was defined as failure to achieve 0% occlusion of target arteries after revascularization was deemed appropriate and attempted. Type of procedure (PCI vs CABG) and revascularization status were obtained from the patient’s procedural note(s). Transfusion status, TIMI major bleeding status, development of cardiogenic shock, development of heart failure, past medical history, and discharge medications were obtained through the patient’s discharge summary. Changes to prescriptions upon discharge were also collected, including anticoagulants (warfarin, direct thrombin inhibitors, and direct factor Xa inhibitors) and diuretics (carbonic anhydrase inhibitors, loop diuretics, thiazide diuretics, and potassium-sparing diuretics).

### Readmission data

Patients readmitted within 30 days were identified from discharge summaries, including date of and reason for readmission. ICD-10 diagnosis codes were used to determine reasons for hospital readmission and were categorized into 2 clinical groups: cardiac (ie, MI, AFIB, pericarditis, cardiac tamponade, and acute chest pain due to apical akinesis or unresolved coronary artery disease [CAD]) and non-cardiac-related admissions.

## Statistical analysis

### Modeling change in HR over 30 days postdischarge

We used a multivariable general linear mixed-effects model (GLMM) to test the associations between clinical characteristics at admission or upon discharge and change in HR during the 30 days postdischarge. We used this model to (1) derive a general form of change in HR from the individual-specific (within-person) patterns of HR change over time, and (2) examine the heterogeneity in HR patterns over time between individuals (between-person) while testing for the contribution of our chosen clinical variables to this heterogeneity. To account for within-patient correlations of consecutive HRs, we used the heterogeneous autoregressive order 1 covariance structure.^9^ To account for longitudinal within-patient changes in HR postdischarge, we assumed a linear change over time. Compared with the model that assumed linear change over time, a piecewise linear random coefficient model with the 6 linear splines (ie, study days 0–5, 5–10, 10–15, 15–20, 20–25, 25–30) did not significantly improve overall model fit to the data based on reduced Akaike information criteria.^10^

The modeling process consisted of 2 steps. At the first step (model 1), we fit an unconditional model without any predictor or covariates (ie, between-person variables) except for a person-specific intercept (ie, baseline/admission HR) and a person-specific slope of change in HR over time. Coefficients of association derived from this model were used as a base with which to compare with the subsequent models. At the next step (model 2), each potential predictor of HR was entered while controlling for between-person variables including age, sex, race, Medicaid status, BB dose, and CCB dose. These variables were chosen to focus our analysis on modifiable cardiovascular comorbidities and surgical/medical interventions. Because there is well-established evidence showing that BBs and CCBs lower HR in the short and long term, we controlled for these medications to unmask any changes in HR contributed by specific comorbidities, admission characteristics, or other medications.

Each patient’s mean admission HR was calculated by averaging HRs collected between study recruitment and hospital discharge. The number of days between recruitment and discharge, as well as the number of recordings, varied across each patient. All models treated mean admission HR as the patient’s mean daily HR associated with study “day” 0 (defined as the inpatient time period, with study day 1 being the day after discharge). We first tested if potential predictors would be associated with their mean admission HR. To compare alternative means of assessing patients’ HR at baseline, we replaced the mean admission HR with the mean HR on the day of discharge and reran the analysis. We then included an interaction term between potential predictors and time within model 2 and tested if it would be associated with the rate of HR change over time. Interactions between covariates were also tested by including the cross-product terms in the model. Differences with 2-sided *P* values <.05 were considered statistically significant. All analyses were performed using STATA version 15.1 (StataCorp, College Station, TX).

From the clinical characteristics mentioned previously, a subset was chosen and analyzed based on clinical relevancy in similar prior studies,^11^^,^^12^ sample size considerations, and multicollinearity testing. These variables were (1) medical history of hypertension, T2DM, prior MI, prior PCI, prior CABG, stroke, dyslipidemia and depression, and any smoking history; (2) admission characteristics: diagnosis of ST-elevated myocardial infarction, LVEF <40%, CABG and/or PCI performed during hospitalization, transfusions performed, development of heart failure, incomplete revascularization; and (3) non-BB/CCB medications: angiotensin-converting enzyme inhibitors and angiotensin II receptor blockers, anticoagulants, diuretics, and adenosine diphosphate receptor inhibitors.

To comment on how missing data were treated, GLLMs can account for unbalanced data sets such as ours by using likelihood-based analyses that provide unbiased estimates under a missing-at-random assumption. To explore whether this unbalanced data set can be considered missing-at-random, we conducted a sensitivity analysis using a random-slope missing-not-at-random mechanism where the likelihood of dropping out is related to the patient’s unobserved slope. This mechanism was fitted through a joint model that combined a survival model for the dropout process and a GLMM for the longitudinal HR outcome, allowing dropout to be related to the patients.^13^ The coefficient estimates for the effect of our variables of interest on change in HR, comparing GLMMs and the joint models, can be found in Supplemental Table 3. The analysis under missing-not-at-random did not materially change the estimates from GLMMs, providing further support for model validity under this study context.

### Descriptive statistics for readmission data and HR distribution

To further assess patterns of HR in relation to readmission, we calculated the mean, range, and frequency of HR recordings across 7 days prior to readmission, as well as the frequency of HRs over 90 bpm, HRs under 60 bpm, and the number of patients represented by those recordings. This threshold was decided based on previous studies, which have shown that admission HRs over 90 bpm or under 60 bpm were associated with increased mortality within 30 days postdischarge.^4^^,^^5^

## Results

### Study population characteristics

**Figure 3:**
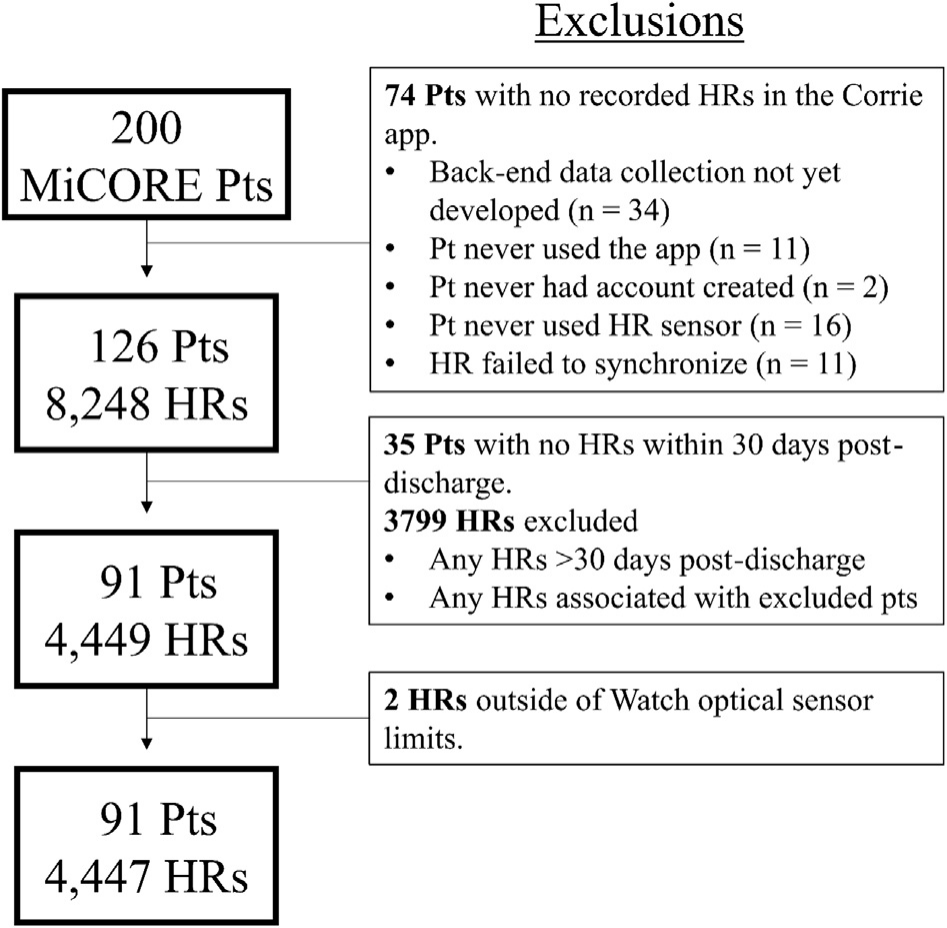
Flow chart of study patient population. HR = heart rate; Pts = patients.

The study population initially included 126 patients from the MiCORE cohort who had 8248 HR recordings. After exclusion of patients and HRs based on our selection criteria detailed in Figure 3, the final cohort included 91 patients with 4447 HR recordings, yielding 1511 calculations of mean daily HR. The baseline characteristics of patients and their associated HR distribution are summarized in Table 1, most notably a mean patient age of 57.0 (SD 10.6), 24 of 91 (26%) female, 63 of 91 (69%) identified as white, 47 of 91 (52%) with hyperlipidemia, 58 of 91 (64%) with hypertension, 33 of 91 (35%) with T2DM, and 6 of 91 (6.6%) who had undergone a CABG at a historical admission. Compared to patients who were excluded from the study (those with no recorded HRs or HRs in the defined study period), patients included in the study were less likely to have a prior MI and were more likely to have complete revascularization during admission (Supplemental Table 4). There was no statistical difference in the prevalence of aggregated cardiovascular risk factors between both groups. This aggregation included patients who had hypertension, T2DM, prior MI, prior stroke, or dyslipidemia as defined by AHA guidelines.^14^ A total of 7.89% of recordings were obtained during admission (day 0), 35.19% between study days 1 and 10, 31.84% between days 11 and 20, and 25.07% between days 21 and 30. Further segmentation of HR data can be found in Supplemental Table 5.

**Table 1:** Baseline characteristics of study patients

Patient characteristics	Patients (N = 91)	HRs (N = 4447)
Demographics		
Age, mean (SD)	57.0 (10.6)	
18–49, n (%)	25 (27.47)	1205 (27.10)
50–64, n (%)	41 (45.05)	3242 (72.90)
≥65, n (%)	25 (27.47)	1004 (22.58)
Female, n (%)	24 (26.37)	1038 (23.34)
White, n (%)	63 (69.23)	3292 (74.03)
BMI, mean (SD)	30.3 (5.0)	
<25, n (%)	8 (8.79)	381 (8.57)
25–35, n (%)	68 (74.73)	3296 (74.12)
>35, n (%)	15 (16.48)	770 (17.32)
Current/previous smoker, n (%)	46 (50.55)	2387 (53.68)
Medicaid, n (%)	7 (7.69)	195 (4.38)
Admission characteristics, n (%)		
STEMI	41 (45.05)	2348 (52.80)
LVEF <40%	16 (17.58)	712 (16.01)
Incomplete revascularization	9 (9.89)	498 (11.20)
PCI performed	69 (75.82)	3484 (78.34)
CABG performed	20 (21.98)	706 (15.88)
Transfusion performed	16 (17.58)	562 (12.64)
TIMI major bleeding	1 (1.10)	8 (0.18)
Development of heart failure	11 (12.09)	397 (8.93)
Cardiac rehab referral	76 (83.52)	3877 (87.18)
Cardiogenic shock	0	0
Past medical history, n (%)		
Hyperlipidemia	47 (51.65)	2332 (52.44)
Hypertension	58 (63.74)	2736 (61.52)
Heart failure†	2 (2.20)	159 (3.58)
Previous PCI	13 (14.29)	668 (15.02)
Previous CABG	6 (6.59)	398 (8.95)
Stroke	6 (6.59)	223 (5.01)
Cerebrovascular disease	2 (2.20)	6 (0.13)
Atrial fibrillation	3 (3.30)	159 (3.58)
Peripheral vascular disease	4 (4.40)	134 (3.01)
Prior MI	9 (9.89)	523 (11.76)
T2DM	33 (36.26)	1450 (32.61)
Chronic lung disease	11 (12.09)	609 (13.69)
Dialysis	1 (1.10)	112 (2.52)
GU/GI bleeding in last 6 mo	1 (1.10)	112 (2.52)
Depression	6 (6.59)	99 (2.23)
Cardiac medications, n (%)		
ADP receptor inhibitors	81 (89.01)	4165 (93.66)
ACE inhibitors/ARBs	52 (57.14)	2699 (60.69)
Anticoagulants	13 (14.29)	453 (10.19)
Aspirin	84 (92.31)	4127 (92.80)
Beta blockers	85 (93.41)	4144 (93.19)
Calcium channel blockers	8 (8.79)	302 (6.79)
Diuretics	28 (30.77)	1007 (22.64)
Statins	89 (97.80)	4356 (97.95)

ACE = angiotensin-converting enzyme; ADP = adenosine diphosphate; ARBs = angiotensin II receptor blockers; BMI = body mass index; CABG = coronary artery bypass graft; GI = gastrointestinal; GU = genitourinary; HR = heart rate; LVEF = left ventricular ejection fraction; MI = myocardial infarction; PCI = percutaneous coronary intervention; STEMI = ST-elevated myocardial infarction; TIMI = thrombolysis in myocardial infarction; T2DM = type 2 diabetes mellitus.

† Heart failure diagnosis prior to index admission.

### Change in HR over 30 days postdischarge

**Figure 4:**
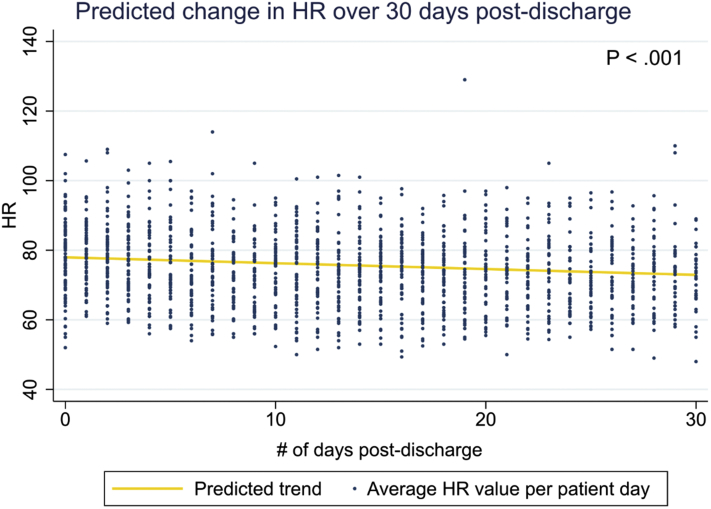
Predicted change in heart rate (HR) across 30 days using unconditional model 1.

Starting at a mean admission HR of 78.0 bpm (95% CI 76.1 to 79.8), each consecutive study day was associated with a 0.172 bpm decrease (*P* < .001, -0.265 to -0.078) in mean daily HR in unconditional model 1 (Figure 4), which remained significant after adjusting for sex, age, race, Medicaid status, and prescription of BBs and CCBs. When examining HRs segmented by time of day, both the morning (-0.151 bpm/day, -0.252 to -0.050) and evening (-0.225 bpm/day, -0.318 to -0.132) recordings showed a progressive decrease in HR per study day. The noon and afternoon recordings showed a similar trend without statistical significance (Supplemental Table 6).

### Associations of risk factors with change in average daily HR across 30 days postdischarge

In fully adjusted model 2 accounting for age, sex, race, Medicaid status, BB dose, and CCB dose, clinical characteristics associated with an elevated mean daily HR during admission included having a prior CABG before admission (8.98 bpm; *P* = .017; 1.59–16.37), depression (12.5 bpm; *P* = .003; 4.29–20.6), discharge prescription for anticoagulants (9.6 bpm; *P* = .001; 4.11–15.16), and discharge prescription for diuretics (5.6 bpm; *P* = .006; 1.60–9.61) compared to those who did not have such characteristics. These results are reflected in Table 2. When the mean admission HR were replaced with the mean HR on the day of discharge, the association estimates were similar.

**Table 2:** Association of clinical characteristics with average admission heart rate and change in average daily heart rate across 30 days postdischarge (in comparison with patients without the designated characteristic)

Clinical characteristics	Relative diff. in mean admission HR	95% CI	*P*	Relative diff. in mean daily HR change	95% CI	*P*
**Medical history**						
Current/previous smoker	0.202	-3.33 to 3.73	.911	0.001	-0.183 to 0.185	.990
Hypertension	-3.18	-7.05 to 0.690	.107	0.190	0.002 to 0.378	.048
T2DM†	1.21	-2.31 to 4.72	.500	0.220	0.044 to 0.395	.014
Prior MI	2.67	-3.65 to 8.98	.409	0.184	-0.109 to 0.477	.219
Prior CABG‡	8.98	1.59 to 16.37	.017	0.255	-0.043 to 0.552	.093
Stroke	1.10	-7.28 to 9.47	.797	0.229	-0.127 to 0.585	.207
Hyperlipidemia	0.125	-3.64 to 3.89	.948	0.179	0.002 to 0.355	.047
Depression	12.5	4.29 to 20.6	.003	0.319	-0.155 to 0.793	.187
**Admission characteristics**						
Diagnosis of STEMI	0.984	-2.77 to 4.73	.607	-0.067	-0.251 to 0.118	.479
LVEF <40%	2.89	-2.62 to 8.39	.304	0.039	-0.201 to 0.279	.753
CABG performed	2.41	-2.31 to 7.13	.317	0.049	-0.198 to 0.296	.697
PCI performed	-2.04	-6.69 to 2.62	.391	-0.066	-0.294 to 0.163	.573
Transfusion performed	-0.026	-5.76 to 5.71	.993	0.068	-0.182 to 0.317	.595
Incomplete revascularization	0.408	-6.37 to 7.19	.906	-0.025	-0.334 to 0.284	.875
Development of heart failure	2.50	-3.47 to 8.47	.412	0.287	-0.002 to 0.576	.052
**Non-BB/CCB medications**						
ACE inhibitors/ARBs	-0.551	-4.74 to 3.64	.797	-0.007	-0.194 to 0.180	.942
Anticoagulants	9.64	4.11 to 15.16	.001	-0.253	-0.517 to 0.010	.059
Diuretics	5.61	1.60 to 9.61	.006	0.096	-0.109 to 0.302	.358
ADP receptor inhibitors	2.06	-4.17 to 8.29	.517	-0.234	-0.590 to 0.123	.198

BB = beta-blocker; CCB = calcium channel blocker; other abbreviations as in Table 1.

† After adjusting for CABG in T2DM association analysis.

‡ After adjusting for T2DM in CABG association analysis.

**Figure 5:**
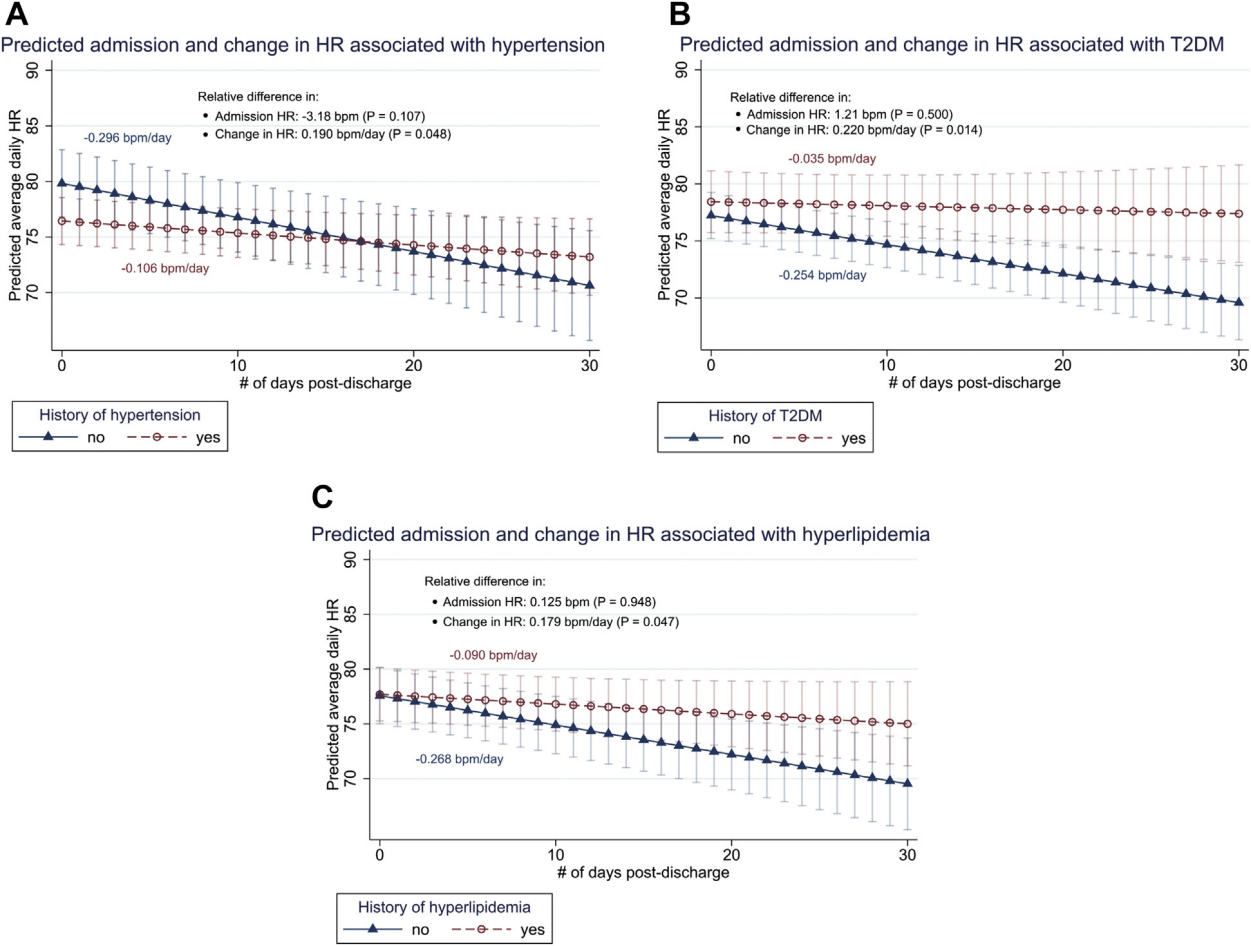
Predicted admission and change in heart rate (HR) over 30 days using fully adjusted model 2 for patients with history of hypertension, type 2 diabetes mellitus (T2DM), or hyperlipidemia.

With respect to change in HR over time (Table 2), patients with hypertension had a -0.11 bpm/day change in HR, while those without hypertension had a -0.30 bpm/day change, demonstrating a slower magnitude of decrease associated with hypertension (0.19 bpm/day difference; *P* = .048; 0.002–0.38). Similarly, patients with T2DM had a slower magnitude of HR decrease compared to their counterparts: -0.04 bpm/day for T2DM compared to -0.25 bpm/day for patients without T2DM (0.22 bpm/day difference; *P* = .014; 0.04–0.40). Patients with hyperlipidemia also had a slower magnitude of HR decrease: -0.09 bpm/day for patients with hyperlipidemia compared to -0.27 bpm/day for patients without (0.18 bpm/day difference; *P* = .047; 0.002–0.36). These comparisons are visualized in Figure 5. The results pertaining to T2DM were obtained after adjusting for potential collinearity with prior CABG.

### HR recordings among patients with 30-day postdischarge readmissions

Eleven out of the 91 patients were readmitted within 30 days postdischarge and included in this subanalysis, with a mean time to readmission of 6.91 days. A description of their demographics and clinical characteristics can be found in Supplemental Table 7. These readmitted patients contributed 364 HR recordings, representing 8.19% of total HRs recorded. One hundred seventy-nine of those HRs were recorded on the day of or day prior to readmission. Five of the readmitted patients did not have any HR recordings within 7 days prior to admission. Table 3 summarizes the HR recordings across 7 consecutive days before readmission. Of note, the mean of HRs recorded, and instances of HR >90 bpm, were highest within 3 days prior to readmission, with variable contributions from each patient. There were no recorded episodes of bradycardia 7 days prior to readmission.

**Table 3:** Descriptive statistics of HR recordings before readmission

	Number of HR Recordings, Mean, and Range		
	# of recordings	Mean, bpm	Range, bpm
Days prior			
1	13	81.2	60-137
2	13	82.6	61-98
3	10	77.3	61-94
4	11	69.2	60-81
5	9	71.4	60-91
6	8	68.9	60-82
7	10	69.6	61-105

	# of HRs > 90 bpm		
	# of recordings	# of pts represented	Cumulative tally of unique pts represented
Days prior			
1	2	1	1
2	7	4	5
3	3	3	6
4	0	0	6
5	1	1	6
6	0	0	6
7	1	1	6

## Discussion

### Principal results

In this prospective study of recovering AMI patients, we used mobile health technologies to determine predictors for change in HR within 30 days postdischarge. No prior studies have examined changes in subacute HR post-AMI outside the hospital setting. We observed that there was a slight decline in mean daily HR over 30 days in both unconditional and adjusted models, which could reflect gradual cardiac recovery secondary to a combination of medical and surgical interventions. Consequently, deviation from this trend, such as a persistently elevated HR, should be studied as a potential biometric of comorbidities contributing to a more difficult recovery process. We identified several putative factors (comorbidities and cardiac medications) associated with increased admission HR and/or deviations in postdischarge HR compared to the overall population. Specifically, a prior history of CABG, history of depression, discharge anticoagulants, and discharge diuretics were associated with a higher average admission HR. Patients with hypertension, T2DM, or hyperlipidemia were associated with a slower decrease in HR compared to those without the corresponding condition. Lastly, for a small subset of patients who were readmitted within 30 days, they typically came back within a week after discharge with elevated HR recordings, generally manifesting 3 days prior to readmission.

### Comparison with prior work

Predictors associated with deviations from HR recovery (hypertension, T2DM, hyperlipidemia) as identified in our study have been associated with higher resting HR among patients with stable CAD.^11^^,^^12^ Other predictors explored by these studies (ie, tobacco use, chronic obstructive pulmonary disease) were not associated with changes in HR among subacute post-AMI patients. Several methodological differences exist between our study and others, which include patient population (stable CAD vs post-AMI), the timing and frequency of HR recordings (single vs multiple repeated measures over time), a continuous vs categorical HR outcome, and confounding factors accounted for.^11^^,^^12^ Importantly, we applied a mixed modeling strategy, which was likely to yield more accurate results by leveraging a substantial number of measurements per individual.

Monitoring HR as a biometric indicator for AMI recovery may elucidate the role of sympathetic activation in both increased HR and adverse outcomes of recovering patients. Patients recovering from AMI are at risk for recurrent MI, sudden cardiac death from arrhythmias, new-onset heart failure, and ventricular remodeling owing to the consequences of sympathetic activation.^15^ To explain the observed decrease in HR post-AMI, current guidelines advise prescribing BB therapy to blunt sympathetic activation, leading to better clinical outcomes.^16^ Additionally, studies have shown that successful reperfusion through PCI resulted in a significant recovery in HR variability, a marker for sympathetic withdrawal and restoration of autonomic balance.^17^ Biometric outcomes post-CABG are more complex: HR variability tends to worsen immediately post-CABG owing to highly invasive surgical manipulation but recovers over several months.^18^ Beyond medical/surgical management, additional guidelines focus on the importance of discharge preparation, which includes referral to an exercise-based cardiac rehabilitation program, reviewing barriers toward medication adherence, education about lifestyle modifications, and a clear follow-up plan.^19^ These components are expected to help decrease HR to cardioprotective ranges over time. Patients in this study also had access to the Corrie smartphone application, which aided in these objectives during their hospitalization and 30 days postdischarge. While we assumed a linear relationship between change in HR over time postdischarge, it is possible that HR might decrease more rapidly in the first 1 or 2 weeks postdischarge owing to a greater adherence to BB therapy or immediate effects of surgical intervention, and then stabilize, reflecting a nonlinear change in HR. While we were able to demonstrate comparable fit between our linear model and a piecewise linear mixed-effects model, future analysis powered by larger sample sizes may better reflect more complex, nonlinear changes during different time points. Further research is also needed to explore the impact of Corrie on HR change between those with and without such intervention.

Diving deeper into this decrease in postdischarge HR, we found a significant decrease in morning and evening HR, but not in noon and afternoon HR. To explain these temporal associations, we have hypothesized 3 potential explanations: (1) direct influence of circadian-based cardiovascular changes on HR reduction, (2) the timing of patient medication intake, and (3) the increased levels of activity midday (for noon and afternoon HRs). Several studies have shown that a patient’s chronotype (a patient’s natural sleep-wake cycle) can contribute to their HR variation and cardiovascular processes such as endothelial vasodilation.^20^^,^^21^ However, these specific mechanisms warrant further research to explain specific trends in HR decline. Regarding medications, most of the patients in this study were prescribed a 1-time-daily-dose BB at discharge. It is possible that most patients had taken their medications either in the morning or in the evening, leading to more immediate declines in HR during those times. Lastly, patients in this cohort had a slightly higher noon and afternoon mean HR, which may reflect increased activity midday. This could lead to an attenuation of HR change, yielding nonsignificant results for noon and afternoon HR recordings.

There are several hypotheses explaining why patients with a prior CABG may have elevated HR during admission. The most likely explanation is that having a prior CABG indicates a patient’s poor health status and extensive CAD. Such a procedure is usually reserved for those with triple vessel disease or left main disease, and for those for whom PCI or medical therapy is not amenable. This chronic disease burden is highlighted by a population-based study from the Veterans Affairs Surgical Quality Improvement Program, showing an increased prevalence of obesity, T2DM, left main CAD, and advanced heart failure among veterans undergoing CABG.^22^ Similarly, our study found that patients with a prior CABG had more comorbidities (6.2 vs 2.0) and were discharged on more medications (13.5 vs 9.7) compared to those without such history. A more detailed analysis of these patients can be found in Supplemental Table 8. Our study contributes to existing literature suggesting that patients with prior CABG tend to have a greater chronic disease burden and may be more susceptible to certain complications.^23^^,^^24^

Patients with either hypertension or T2DM have a slower rate of HR decrease compared to patients without the comorbidity. The short-term relationship observed in recovering AMI patients is comparable to the results of previous studies that focused on long-term HR changes in relation to these comorbidities in the general population.^12^^,^^25^ Elevated HR is a common feature in patients with hypertension and has been implicated in its pathogenesis and progression.^26^^,^^27^ Hypertension has also been shown to cause impairments in autonomic cardiovascular control, such as abnormalities in baroreceptor and chemoreceptor signaling, leading to reductions in parasympathetic tone.^28^ Patients with T2DM have been shown to derive more myocardial energy from nonesterified fatty acids, resulting in higher myocardial oxygen consumption and a higher resting HR.^12^ When coupled with an acute myocardial insult or chronic CAD leading to chronotropic incompetence, diabetic patients were more likely to suffer an adverse cardiac event.^29^ For both the hypertensive and diabetic patient populations, the slower rate of HR recovery may be partially attributable to an escalation in sympathetic activity and reflect barriers toward HR recovery. In addition, chronic sympathetic overactivity may affect glucose tolerance and worsen a patient’s glucose control.^12^ The exact relationship between HR, BP, and the role of sympathetic activation is still an active area of discussion, especially in the context of obesity, T2DM, and metabolic syndrome.^27^

Patients with a history of hyperlipidemia also showed a slower rate of HR decrease in this study. Prior research has shown positive associations between HR and hyperlipidemia, which includes elevated serum total cholesterol, LDL cholesterol, and triglyceride levels through blood screening as well as through documentation.^30^^,^^31^ The mechanism is likely multifactorial, involving development of CAD and endothelial dysfunction leading to impaired autonomic regulation over long-term cumulative exposure.^32^, ^33^, ^34^ However, the short-term impact of hyperlipidemia on HR change during a patient’s subacute recovery period remains unclear. Furthermore, this association pertains more generally to a prior documentation of hyperlipidemia, as we did not record patients’ current lipid status obtained through bloodwork. Further interpretation of this association would require more in-depth analysis of patients’ current lipid status based on laboratory work and usage of lipid-lowering medications.

We also identified an association of prescription of diuretics with increased HR during admission, but not with change in HR. This association can be partly explained from the patient’s cardiac status and medication’s mechanism of action. Indications for discharge diuretics post-AMI include volume overload and acute heart failure during admission,^16^ consistent with our study findings after reviewing each patient’s indication for diuretics (Supplemental Table 9). In the acute setting, this complication leads to a decrease in blood pressure owing to reduced cardiac contractility, leading to a compensatory increase in HR to preserve cardiac output. This increase in HR has also been shown in patients with heart failure, both for discharge HR and for first follow-up HR 1 week postdischarge.^35^^,^^36^ While these discharge medications may indirectly reflect the impact of underlying disease burden on AMI recovery, it is difficult to make robust conclusions owing to the variability in medical optimization and treatment plan during admission.

Similarly, patients discharged with anticoagulant therapy had an elevated admission HR but not change in HR. Current guidelines indicate anticoagulative therapy among patients with AFIB, deep venous thrombosis, left ventricular thrombus, and other hypercoagulable disorders for at least 3 months.^37^ Thus, it is likely that prescription of anticoagulant therapy is an indicator of adverse events occurring during admission, such as occurrence of AFIB, which is characterized by rapid, irregular atrial contractions leading to uncoordinated ventricular contractions.

While past studies have speculated an association between depression and increased admission HR owing to impaired autonomic function in patients (especially those with concurrent CAD),^38^ data from a large population-based prospective cohort study did not support a causal pathway or genetic pleiotropy that explained a putative link between depression and autonomic dysregulation.^39^ Alternatively, it is possible that certain classes of antidepressants could confound this association.^39^ On chart review, 13% of patients (10 out of 77 from Johns Hopkins recruitment sites) were prescribed antidepressants, which include selective serotonin reuptake inhibitors, serotonin-noradrenaline reuptake inhibitors, tricyclic antidepressants, atypical antidepressants, and antiepileptics used for refractory depressions. Further research focusing on the impact of these antidepressants is warranted to disentangle this relationship. This limitation, along with the small sample size of patients, warrants further exploration into depression as well as other psychiatric diagnoses on post-AMI recovery.

In our analysis of readmissions, more than half of these patients exhibited an elevation in HR greater than 90 bpm within 3 days of readmission. However, the small sample size prohibits us from any firm interpretation of the current findings. Apart from using an HR threshold as a surrogate marker of signs of decompensation, future research may investigate acute changes in HR within various time frames before readmission. It may also investigate the effectiveness of real-time telemedicine interventions when higher HR values are detected.

### Limitations

One limitation affecting the applicability of this study is that our patients had access to a digital health intervention, making our study population less representative of all post-AMI patients. Self-directed patient participation may introduce selection bias as another limitation of this study. Patients who were healthier during their admission may have more physical and mental bandwidth to engage in a new technology. As such, having a healthier sample is more likely to attenuate current results than produce biased estimates: elevations in HR may be underestimated, as healthier patients tend to have better autonomic control and hemodynamic stability. Further evidence of selection bias includes a decreased prevalence of prior MI for patients included in this study. Another limitation is the small sample size in the current analysis, which particularly affected the reliability of the association estimate for prior CABG, given that only 6 patients had prior CABG. There were also insufficient parameters to help differentiate resting and exercise-induced HR through the Apple Watch. While AMI patients are advised to limit heavy exertion postdischarge, elevated HR due to increased activity still represents a confounding factor that reflects limitations of the Apple Watch.

Our statistical model did not account for medication titrations that occurred during follow-up within the study period. To evaluate the impact of these changes, we conducted a chart review of 77 of 91 available patient records at Johns Hopkins (Supplemental Table 10) and found that 20% of patients had a CV medication changed during follow-up, and 14% had a BB/CCB changed. Fourteen percent of HRs were collected after a CV medication change and 7% of HRs were collected after a BB/CCB change. While these titrations had an impact on a minority of HR recordings predominantly 2 weeks after discharge, they are a limitation to the study. Another limitation is the absence of reliable medication adherence data among patients. While there are data available through manual chart review associated with patient follow-up visits, it would not be appropriate to confidently reference these statistics without further evaluation of data accuracy and potential reporting bias.

### Clinical implications

Patients recovering from AMI are particularly vulnerable during their subacute period and require personalized rehabilitation. Historically, HR and other vital signs during follow-up have often been used as isolated snapshots of a patient’s wellbeing. The lack of multiple data points over time limits evaluation of trends necessary to monitor patient recovery status. The introduction of wearables such as the Apple Watch facilitates longitudinal monitoring of vital signs and can aggregate larger quantities of real-time health data without requiring frequent clinic visits.

This continuous source of data allows us to better understand each patient’s recovery progress day-by-day, pinpoint higher-risk patients who exhibit HR trends that deviate from the norm, and monitor potential deviations in patient behavior. Our study highlights the overall decrease in HR post-AMI (0.2 bpm/day) over 30 days postdischarge as a promising indicator of recovery with current guideline-directed therapies and use of digital health technology.^16^ Understanding the predictors of elevated HR within this subacute period helps lay the groundwork for future algorithms that take static inputs (comorbidities, admission data), dynamic inputs (HR, blood pressure, activity, medications prescribed), and their interactions to assess for early signals of decompensation. Future steps include analyzing more granular HR data (beyond 4 measurements per day) and more rigorously exploring biometric data for readmitted patients. Beyond HR data, collecting other biometrics such as blood pressure, step count, daily activity, and sleep time through consumer wearables may be informative. There also exists an opportunity to investigate sustained bradycardia, which has been linked to excess morbidity and mortality.^5^ Other admission variables, such as occurrences of postprocedural AFIB or other arrhythmias, may also be collected to elaborate on factors affecting HR change in the subacute period.

This study is a first step in understanding post-AMI wearable HR data and its future uses in remote patient monitoring leading to targeted early interventions. Prior research has shown the potential role for other forms of remote patient monitoring. One study assessed the 30-day readmission rates of AMI patients who used a telemedicine system consisting of (1) a patient-worn cardio-beeper that can transmit 12-lead electrocardiograms, (2) a 24-hour-a-day call center, and (3) a mobile intensive care unit. Out of 897 patients, 5.8% were readmitted within 30 days, compared to readmission rates of 11%–28% in the region. Solutions such as this highlight how wearables can be incorporated into larger workflows to streamline medical decision-making and provide numerous capabilities as a longitudinal research tool. Furthermore, their financial feasibility is highlighted by recent policy changes by the Centers for Medicare & Medicaid Services, which has helped broaden reimbursement for digital health tools involved in remote patient monitoring.^40^^,^^41^

## Conclusion

In this study, we characterized longitudinal HR and predictors of HR change in recovering AMI patients to investigate its potential as a biometric for recovery. We found that these patients have a decline in average daily HR of 0.2 bpm/day during their 30-day postdischarge period. Patients’ past medical history, including prior CABG, hypertension, T2DM, hyperlipidemia, depression, and specific cardiac medications prescribed at discharge, may contribute to the variation in admission HR and/or the rate of HR change between patients postdischarge. Patients readmitted within 30 days postdischarge showed elevated HR recordings within 3 days prior to admission, based on a limited data set of 11 participants. This study highlights the feasibility of analyzing large quantities of longitudinal data collected from a commercial wearable and digital health application in estimating postdischarge HR trends among AMI patients. Ultimately, this enables us to monitor HR outside the hospital and better contextualize HR data based on the patient’s clinical profile, and could further enable precision medicine by guiding more targeted treatments at the appropriate times.

## References

[1] New Y, Health H, Corporation S, & Core EY. (2016). Variation in 30-day mortality rates across hospitals following hospital admission for acute myocardial infarction, chronic obstructive pulmonary disease, heart failure, pneumonia, and acute ischemic stroke. (July 2013), 2016–2017.

[2] Reindl M., Reinstadler S.J., Feistritzer H.-J. (2016). Heart rate and left ventricular adverse remodelling after ST-elevation myocardial infarction. Int J Cardiol.

[3] Xu T., Zhan Y., Xiong J. (2016). The relationship between heart rate and mortality of patients with acute coronary syndromes in the coronary intervention era: meta-analysis. Medicine (Baltimore).

[4] Asaad N., El-Menyar A., AlHabib K.F. (2014). Initial heart rate and cardiovascular outcomes in patients presenting with acute coronary syndrome. Acute Card Care.

[5] Kovar D., Cannon C.P., Bentley J.H., Charlesworth A., Rogers W.J. (2004). Does initial and delayed heart rate predict mortality in patients with acute coronary syndromes?. Clin Cardiol.

[6] Falter M., Budts W., Goetschalckx K., Cornelissen V., Buys R. (2019). Accuracy of Apple Watch measurements for heart rate and energy expenditure in patients with cardiovascular disease: cross-sectional study. JMIR mHealth uHealth.

[7] Spaulding E.M., Marvel F.A., Lee M.A. (2019). Corrie Health digital platform for self-management in secondary prevention after acute myocardial infarction. Circ Cardiovasc Qual Outcomes.

[8] Apple: Monitor your heart rate with Apple Watch [Internet]. Apple. 2021. Available from: https://support.apple.com/en-us/HT204666.

[9] Madden J.M., Li X., Kearney P.M., Tilling K., Fitzgerald A.P. (2017). Exploring diurnal variation using piecewise linear splines: an example using blood pressure. Emerg Themes Epidemiol.

[10] Akaike H. (1974). A new look at the statistical model identification. IEEE Trans Automat Contr.

[11] Lorgis L., Zeller M., Jourdain P. (2009). Heart rate distribution and predictors of increased heart rate among French hypertensive patients with stable coronary artery disease. Data from the LHYCORNE cohort. Arch Cardiovasc Dis.

[12] Ergene O., Akyildiz Z.I., PULSE Study Group (2014). Analysis of resting heart rate and clinical characteristics in outpatients with stable coronary artery disease in Turkey: PULSE study. Cardiol J.

[13] Magnusson K. Methodological issues in psychological treatment research: applications to gambling research and therapist effects. 2019.

[14] Arnett D.K., Blumenthal R.S., Albert M.A. (2019). 2019 ACC/AHA Guideline on the Primary Prevention of Cardiovascular Disease: A Report of the American College of Cardiology/American Heart Association Task Force on Clinical Practice Guidelines. Circulation.

[15] Brieger D., Fox K.A.A., FitzGerald G. (2009). Predicting freedom from clinical events in non-ST-elevation acute coronary syndromes: the Global Registry of Acute Coronary Events. Heart.

[16] O’Gara P.T., Kushner F.G., Ascheim D.D. (2013). 2013 ACCF/AHA guideline for the management of ST-elevation myocardial infarction: a report of the American College of Cardiology Foundation/American Heart Association Task Force on Practice Guidelines. Circulation.

[17] Abdelnaby M.H. (2018). Effect of percutaneous coronary intervention on heart rate variability in coronary artery disease patients. Eur Cardiol.

[18] Lakusic N., Mahovic D., Kruzliak P., Cerkez Habek J., Novak M., Cerovec D. (2015). Changes in heart rate variability after coronary artery bypass grafting and clinical importance of these findings. Biomed Res Int.

[19] Mercado M.G., Smith D.K., McConnon M.L. (2013). Myocardial infarction: management of the subacute period. Am Fam Physician.

[20] Facer-Childs E.R., Pake K., Lee V.Y., Lucas S.J.E., Balanos G.M. (2019). Diurnal variations in vascular endothelial vasodilation are influenced by chronotype in healthy humans. Front Physiol.

[21] Dunn J.S., Taylor C.E. (2014). Cardiovascular reactivity to stressors: effect of time of day?. Chronobiol Int.

[22] Cornwell L.D., Omer S., Rosengart T., Holman W.L., Bakaeen F.G. (2015). Changes over time in risk profiles of patients who undergo coronary artery bypass graft surgery: the Veterans Affairs Surgical Quality Improvement Program (VASQIP). JAMA Surg.

[23] Jneid H., Addison D., Bhatt D.L. (2017). 2017 AHA/ACC Clinical Performance and Quality Measures for Adults With ST-Elevation and Non-ST-Elevation Myocardial Infarction: A Report of the American College of Cardiology/American Heart Association Task Force on Performance Measures. J Am Coll Cardiol.

[24] Kosmidou I., Chen S., Kappetein A.P. (2018). New-onset atrial fibrillation after PCI or CABG for left main disease: the EXCEL trial. J Am Coll Cardiol.

[25] Seabra-Gomes R. (2010). Investigadores do Registo PULSAR: Characterization of an ambulatory population with stable coronary artery disease and importance of heart rate: the PULSAR registry. Rev Port Cardiol [Internet].

[26] Reule S., Drawz P.E. (2012). Heart rate and blood pressure: any possible implications for management of hypertension?. Curr Hypertens Rep.

[27] Palatini P. (2011). Role of elevated heart rate in the development of cardiovascular disease in hypertension. Hypertension.

[28] Grassi G., Ram V.S. (2016). Evidence for a critical role of the sympathetic nervous system in hypertension. J Am Soc Hypertens.

[29] Zafrir B., Azencot M., Dobrecky-Mery I., Lewis B.S., Flugelman M.Y., Halon D.A. (2016). Resting heart rate and measures of effort-related cardiac autonomic dysfunction predict cardiovascular events in asymptomatic type 2 diabetes. Eur J Prev Cardiol.

[30] Ehrenwald M., Wasserman A., Shenhar-Tsarfaty S. (2019). Exercise capacity and body mass index - important predictors of change in resting heart rate. BMC Cardiovasc Disord.

[31] Kristal-Boneh E., Harari G., Green M.S. (1994). Serum lipids and haematological factors associated with resting heart rate: the CORDIS Study. Cardiovascular Occupational Risk Factors Detection in Israeli Industries. J Cardiovasc Risk.

[32] Vogel R.A. (1997). Coronary risk factors, endothelial function, and atherosclerosis: a review. Clin Cardiol.

[33] Stapleton P.A., Goodwill A.G., James M.E., Brock R.W., Frisbee J.C. (2010). Hypercholesterolemia and microvascular dysfunction: interventional strategies. J Inflamm.

[34] Barreto-Filho J.A.S., Consolim-Colombo F.M., Guerra-Riccio G.M. (2003). Hypercholesterolemia blunts forearm vasorelaxation and enhances the pressor response during acute systemic hypoxia. Arterioscler Thromb Vasc Biol.

[35] Kim T.-H., Kim H., Kim I.-C. (2018). Heart rate at first postdischarge visit and outcomes in patients with heart failure. Heart.

[36] DeVore A.D., Mi X., Mentz R.J. (2016). Discharge heart rate and β-blocker dose in patients hospitalized with heart failure: findings from the OPTIMIZE-HF registry. Am Heart J.

[37] January C.T., Wann L.S., Calkins H. (2019). 2019 AHA/ACC/HRS Focused Update of the 2014 AHA/ACC/HRS Guideline for the Management of Patients With Atrial Fibrillation: A Report of the American College of Cardiology/American Heart Association Task Force on Clinical Practice Guidelines and the Heart Rhythm Society in Collaboration With the Society of Thoracic Surgeons. Circulation.

[38] Aydin Sunbul E., Sunbul M., Gulec H. (2017). The impact of major depression on heart rate variability and endothelial dysfunction in patients with stable coronary artery disease. Gen Hosp Psychiatry.

[39] Hu M.X., Milaneschi Y., Lamers F. (2019). The association of depression and anxiety with cardiac autonomic activity: the role of confounding effects of antidepressants. Depress Anxiety.

[40] Dey P., Jarrin R., Mori M., Geirsson A., Krumholz H.M. (2021). Leveraging remote physiologic monitoring in the COVID-19 pandemic to improve care after cardiovascular hospitalizations. Circ Cardiovasc Qual Outcomes.

[41] Mecklai K., Smith N., Stern A.D., Kramer D.B. (2021). Remote patient monitoring — overdue or overused?. N Engl J Med.

