# Supplementary material for: Heart rate trajectories in patients recovering from acute myocardial infarction: A longitudinal analysis of Apple Watch heart rate recordings
Source: Cardiovasc Digit Health J. 2021 May 12;2(5):270–81. doi: 10.1016/j.cvdhj.2021.05.003 (PMC8890343; doi:10.1016/j.cvdhj.2021.05.003)
Supplement: Supplemental Tables [file mmc1.docx]

**Supplemental Table 1.** ICD-10 Codes used to adjudicate clinical comorbidities

| Clinical Characteristics | ICD-10 Codes |
| --- | --- |
| STEMI | [I21.0] STEMI of anterior wall  [I21.01] STEMI involving left main coronary artery  [I21.02] STEMI involving left anterior descending coronary artery  [I21.09] STEMI involving other coronary artery of anterior wall  [I21.1] STEMI of inferior wall  [I21.11] STEMI involving right coronary artery  [I21.19] STEMI involving other coronary artery of inferior wall  [I21.2] STEMI of other sites  [I21.21] STEMI involving left circumflex coronary artery  [I21.29] STEMI involving other sites  [I21.3] STEMI of unspecified site |
| Prior CABG | [Z95.1] Presence of aortocoronary bypass graft |
| Prior PCI | [Z95.5] Presence of coronary angioplasty implant and graft |
| Transfusion History | [Z92.89] Personal history of other medical treatment (specifically labeled for prior transfusion by physician) |
| Hyperlipidemia | [E78.0] Pure hypercholesterolemia  [E78.00] Pure hypercholesterolemia, unspecified  [E78.01] Familial hypercholesterolemia  [E78.1] Pure hyperglyceridemia  [E78.2] Mixed hyperlipidemia  [E78.3] Hyperchylomicronemia  [E78.4] Other hyperlipidemia  [E78.5] Hyperlipidemia, unspecified |
| Hypertension | [I10] Essential (primary) hypertension |
| Heart failure | [I50.1] Left ventricular failure, unspecified  [I50.2] Systolic (congestive) heart failure  [I50.20] Unspecified systolic (congestive) heart failure  [I50.21] Acute systolic (congestive) heart failure  [I50.22] Chronic systolic (congestive) heart failure  [I50.23] Acute on chronic systolic (congestive) heart failure  [I50.3] Diastolic (congestive) heart failure  [I50.30] Unspecified diastolic (congestive) heart failure  [I50.31] Acute diastolic (congestive) heart failure  [I150.32] Chronic diastolic (congestive) heart failure  [I150.33] Acute on chronic diastolic (congestive) heart failure  [I50.4] Combined systolic and diastolic heart failure  [I50.40] Unspecified combined systolic and diastolic heart failure  [I50.41] Acute combined systolic and diastolic heart failure  [I50.8] Other heart failure  [I50.9] Heart failure, unspecified |
| Stroke | [I63] Cerebral infarction (and all subgroups)  [G45.9] Transient cerebral ischemic attack, unspecified |
| Atrial fibrillation | [I48.0] Paroxysmal atrial fibrillation  [I48.1] Persistent atrial fibrillation  [I48.11] Longstanding persistent atrial fibrillation  [I48.19] Other persistent atrial fibrillation  [I48.2] Chronic atrial fibrillation  [I48.20] Chronic atrial fibrillation, unspecified  [I48.21] Permanent atrial fibrillation  [I48.91] Unspecified atrial fibrillation |
| Peripheral Vascular Disease | [I73.9] Peripheral vascular disease, unspecified |
| Myocardial Infarction | [I21] Acute myocardial infarction (and all subgroups) |
| Type 2 Diabetes | [E11] Type 2 diabetes mellitus (and all subgroups) |
| Chronic Lung Disease | [J43] Emphysema (and all subgroups)  [J44] Other chronic obstructive pulmonary disease (and all subgroups)  [J45] Asthma (and all subgroups) |
| GU/GI Bleed in last 6 months | [K29.71] Gastritis, unspecified, with bleeding  [K62.5] Hemorrhage of anus and rectum  [K91.6] Intraoperative hemorrhage and hematoma of a digestive system organ or structure complicating a procedure  [K92.0] Hematemesis  [K92.1] Melena  [K92.2] Gastrointestinal hemorrhage, unspecified  [N93] Other abnormal uterine and vaginal bleeding (and all subgroups)  [R82.3] Hemoglobinuria |
| Depression | [F32] Major depressive disorder, single episode (and all subgroups)  [F33] Major depressive disorder, recurrent (and all subgroups) |
| Cardiogenic Shock  *counted if occurred after study enrollment | [R57.0] Cardiogenic Shock |

**Supplemental Table 2.** Categorizations of primary insurance and racial demographics

| Categorizations | Raw data variables |
| --- | --- |
| Medicaid (yes) | Medicaid |
| Medicaid (no) | Commercial/Preferred Provider organization |
|  | Health maintenance organization |
|  | Medicare |
|  | Self-pay |
| White (yes) | White or Caucasian |
| White (no) | American Indian or Alaska Native |
|  | Asian |
|  | Black or African American |
|  | Declined to Answer |
|  | Native Hawaiian or Other Pacific Islander |
|  | Other |
|  | Unknown |
|  | Hispanic or Latino |

**Supplemental Table 3.** Comparison of GLMMs (generalized linear effects models) with joint model after adjusting for age, sex, and race.

|  | **Age, Sex & Race adjusted Model (Interaction with study day β, SE)** | |
| --- | --- | --- |
| **Variables of interest** | **GLMMs** | **Joint model** |
| Prior CABG | 0.33 (0.18) | 0.27 (0.18) |
| T2DM | 0.27 (0.10) | 0.24 (0.07) |
| Hypertension | 0.19 (0.10) | 0.13 (0.29) |

**Supplemental Table 4.** Comparison of patients included and excluded from the study

|  | | Patients Included (N = 91) | Patients Excluded (N=109) | *P*^†^ |
| --- | --- | --- | --- | --- |
| Demographics | |  |  |  |
|  | Age, mean (SD) | 57.0 (10.6) | 59.0 (12.3) | .225 |
|  | BMI, mean (SD) | 30.3 (5.0) | 30.9 (6.6) | .535 |
|  | Female, n (%) | 24 (26.4) | 34 (31.2) | .455 |
|  | White, n (%) | 63 (69.2) | 74 (67.9) | .839 |
|  | Medicaid, n (%) | 7 (7.7) | 15 (13.8) | .172 |
|  | Current/Previous smoker, n (%) | 46 (50.5) | 64 (58.7) | .248 |
| Past Medical History, n (%) | |  |  |  |
|  | Hypertension | 58 (63.7) | 76 (69.7) | .370 |
|  | T2DM | 33 (36.3) | 51 (46.8) | .133 |
|  | Prior MI | 9 (9.9) | 27 (24.8) | .006 |
|  | Prior CABG | 6 (6.6) | 12 (11.0) | .277 |
|  | Stroke | 6 (6.6) | 9 (8.3) | .656 |
|  | Dyslipidemia | 47 (51.7) | 64 (58.7) | .317 |
|  | Depression | 6 (6.6) | 9 (8.3) | .656 |
|  | CV risk factors^‡^ | 77 (84.6) | 96 (88.1) | .365 |
| Admission Characteristics, n (%) | |  |  |  |
|  | Diagnosis of STEMI | 41 (45.1) | 38 (34.9) | .142 |
|  | LVEF <40% | 16 (17.6) | 26 (23.9) | .278 |
|  | CABG performed | 20 (22.0) | 32 (29.4) | .236 |
|  | PCI performed | 69 (75.8) | 70 (64.2) | .076 |
|  | Transfusion performed | 16 (17.6) | 20 (18.4) | .888 |
|  | Incomplete revascularization | 9 (9.9) | 28 (25.7) | .004 |
|  | Development of heart failure | 11 (12.1) | 16 (14.7) | .593 |

^†^ Calculated using Chi^2^ test for binomial variables, independent t-test for continuous variables

^‡^ CV Risk factors = Hypertension, T2DM, Prior MI, Stroke, Dyslipidemia (smoking not included due to lack of data specificity in smoking quantity and length)

**Supplemental Table 5.** Descriptive statistics of all HR measurements and overall average daily HR per patient

|  | **Total**  **(N=4,447)** | **Morning**  **(N=1,391)** | **Noon**  **(N=1,083)** | **Afternoon (N=982)** | **Evening (N=991)** | **Average Daily**  **(N=1,511)** |
| --- | --- | --- | --- | --- | --- | --- |
|  |  |  |  |  |  |  |
| Mean (SD) | 74.2 (12.6) | 73.8 (13.2) | 74.6 (12.5) | 74.5 (12.4) | 74.2 (12.2) | 74.5 (10.9) |
| Median | 73 | 73 | 73 | 73 | 73 | 74 |
| Interquartile range | 16 | 17 | 17 | 17 | 16 | 15 |
| 5^th^ percentile | 56 | 54 | 57 | 57 | 57 | 57.5 |
| 95^th^ percentile | 97 | 97 | 96 | 97 | 96 | 93.5 |
| Range | 36-155 | 39-137 | 36-155 | 36-133 | 44-139 | 48-129 |

**Supplemental Table 6.** Estimated change in HR over 30 days post discharge

|  | | Mean Daily HR | 95% CI | *P* |
| --- | --- | --- | --- | --- |
| Average Daily HR | |  |  |  |
|  | Univariate (N=91) ^†^ | −0.168 | −0.249 to −0.072 | <.001 |
|  | Adjusted (N=91) ^‡^ | −0.177 | −0.491 to 0.136 | .268 |
| Time of Day HR | |  |  |  |
|  | Morning HR (N=86) | -0.150 | −0.252 to −0.050 | .004 |
|  | Noon HR (N=84) | -0.089 | −0.186 to 0.009 | .075 |
|  | Afternoon HR (N=85) | -0.068 | −0.172 to 0.036 | .198 |
|  | Evening HR (N=73) | -0.217 | −0.308 to −0.125 | <.001 |

^†^ N refers to the number of patients included in the estimated change in HR

^‡^ Adjusted model includes sex, age, white race, Medicaid status, prescription of BB and CCB class + dose combinations

**Supplemental Table 7.** Demographics and clinical characteristics of study patients readmitted within 30 days post discharge

|  | | | Patients readmitted within 30 days (N=11) | Patients not readmitted (N=80) |
| --- | --- | --- | --- | --- |
| Demographics | | |  |  |
|  | Age, mean (SD) | | 56.8 (13.1) | 57 (10.3) |
|  | BMI, mean (SD) | | 31.0 (7.1) | 30.3 (4.7) |
|  | Female, n (%) | | 5 (45) | 19 (24) |
|  | White, n (%) | | 8 (73) | 55 (69) |
| Clinical Characteristics | | |  |  |
|  | Hypertension**,** n (%) | | 9 (82) | 49 (61) |
|  | T2DM**,** n (%) | | 7 (64) | 26 (33) |
|  | Dyslipidemia, n (%) | | 8 (73) | 39 (49) |
|  | Total comorbidities, mean (SD) | | 3.6 (2.7) | 2.1 (1.6) |
|  | Total discharge medications, mean (SD) | | 14.5 (6.3) | 9.4 (3.1) |
| Readmission Criteria, n (%) | | |  |  |
|  | Non-cardiac related | | 6 (55) |  |
|  | **Cardiac related** | | 5 (45) |  |
|  |  | Chest pain (cardiac hypokinesis) | 2 |  |
|  |  | Pericarditis | 1 |  |
|  |  | Cardiac tamponade | 1 |  |
|  |  | New onset AFIB | 1 |  |

**Supplemental Table 8.** Clinical characteristics of patients with history of CABG compared to no history of CABG

|  | | HRs for patients with Hx of CABG (N=398) | HRs for patients with no Hx of CABG (N=4049) |
| --- | --- | --- | --- |
| HR characteristics | |  |  |
|  | Mean HR (SD) | 78.46 (13.32) | 73.83 (12.50) |
|  | Mean daily HR (SD) | 80.64 (11.87) | 73.91 (10.66) |
|  | | **Patients with Hx of CABG (N=6)** | **Patients with no Hx of CABG (N=85)** |
| Patient Characteristics | |  |  |
|  | Age, mean (SD) | 61.3 (13.2) | 56.7 (10.4) |
|  | BMI, mean (SD) | 29.1 (3.4) | 30.4 (5.1) |
|  | T2DM, n (%) | 5 (83) | 28 (33) |
|  | Dyslipidemia, n (%) | 5 (83) | 42 (49) |
|  | Hypertension, n (%) | 5 (83) | 53 (62) |
|  | LVEF < 40%, n (%) | 3 (50) | 13 (15) |
|  | Total comorbidities, mean (SD) | 6.2 (2.7) | 2.0 (1.5) |
|  | Total discharge medications, mean (SD) | 13.5 (4.4) | 9.7 (3.8) |

**Supplemental Table 9. Indications for medication prescription**

|  | | Patients, n (%) |
| --- | --- | --- |
| Diuretics (N=28) | |  |
|  | Heart failure/  Reduced ejection fraction | 10 (36) |
|  | Hypertension | 1 (3.6) |
|  | Hypervolemia | 14 (50) |
|  | Unknown | 3 (11) |
| Anticoagulants (N=13) | |  |
|  | Left ventricular apical akinesis | 5 (38) |
|  | Left ventricular apical thrombus | 1 (7.7) |
|  | Deep vein thrombosis/pulmonary embolism | 3 (23) |
|  | History of AFIB | 3 (23) |
|  | Polycythemia vera | 1 (7.7) |

**Supplemental Table 10.** Patient medication titrations after discharge

|  | # of Patients Reviewed (N=77) ^†^ |
| --- | --- |
| Any CV meds titrated within 30 days post-discharge^‡^ | 20 (26%) |
| Any BB/CCB titration within 30 days | 11 (14%) |
|  | **Average # of days** |
| Until first CV med change | 13.6 |
| Until first BB/CCB change | 13.6 |
|  | **# of HRs recorded (N=3740)** |
| After first CV med change | 510 (14%) |
| After first BB/CCB change | 263 (7%) |

† 77 out of 91 patient charts reviewed from Johns Hopkins Medical Institutions. Unable to review 14 patient charts within Reading and MGH hospital systems

‡ Includes aspirin, ACE-inhibitors/ARBs, BBs, CCBs, anticoagulants, ADP-receptor inhibitors, and diuretics
